# The Deficit of Early Selective Attention in Adults With Sluggish Cognitive Tempo: In Comparison With Those With Attention-Deficit/Hyperactivity Disorder

**DOI:** 10.3389/fpsyg.2021.614213

**Published:** 2021-03-10

**Authors:** Yelin Park, Jang-Han Lee

**Affiliations:** Department of Psychology, College of Social Science, Chung-Ang University, Seoul, South Korea

**Keywords:** sluggish cognitive tempo (SCT), attention-deficit/hyperactivity disorder (ADHD), selective attention, early information processing process, late information processing process, load theory

## Abstract

Sluggish cognitive tempo (SCT) is a cluster of attentional symptoms characterized by slow information processing and behavior, distractibility, mental confusion, absent-mindedness, and hypoactivity. The present study aimed to compare early and late selective attention in the information processing speed of adults with SCT to those with attention-deficit/hyperactivity disorder (ADHD) and adults without any attentional problems. The participants were screened using Barkley Adult ADHD Rating Scale-IV and divided into the following groups: SCT (*N* = 24), ADHD (*N* = 24), and controls (*N* = 25). All participants completed the irrelevant distractor task measuring early and late selective attention under load condition (low vs. high) and distractor condition (no-distractor vs. distractor). The inefficiency index was calculated by subtracting the reaction time of no-distractor condition of correct trials from the reaction time of distractor condition to control the impact of accuracy. Upon analysis, the SCT group showed a lower efficiency compared to the ADHD group under high load, while the ADHD group showed lower efficiency under low load than high load. This meant that the ADHD group had increased efficiency of selective attention with higher load, while the SCT group had low efficiency of selective attention even under high loads. These results suggest that the symptoms of “slow” or “distracted” in SCT could be attributed to the reduced speed and efficiency of selective attention in early information processing and the problem can be pronounced in situations with distractors. The results of the study imply that the attention-deficit-like symptoms shown in those with SCT and ADHD can be distinguished in specific stage of information processing.

## Introduction

Sluggish cognitive tempo (SCT) is an attentional construct defined as a cluster of symptoms characterized by slow behavior, slow information processing, mental confusion, absent-mindedness, and hypoactivity (Barkley, 2012, 2013; Becker and Barkley, 2018). Despite increasing interest in research on cognitive and socioemotional functioning of SCT, there remains a need for research on core cognitive symptoms. Initially, SCT was considered a specifier of attention-deficit/hyperactivity disorder (ADHD) (Garner et al., 2017). However, a growing body of research indicated that SCT is a distinct attentional problem, separate from ADHD, evidenced by differences in cognitive and social functioning, statistical factor analysis, and comorbidity patterns (Jarrett et al., 2017; Smith et al., 2019). While symptoms of being “easily distracted” or “mentally confused” can be observed in both, those with SCT experience problems in perceptual processes, attentional selection, and orienting/shifting of attention rather than problems in executive function (Mikami et al., 2007; Jarrett et al., 2020). Conversely, those with ADHD face problems with executive functions, including response inhibition (Weigard et al., 2018). Thus, it would be helpful to consider the difference in attentional problems in the early and late information processing of SCT and ADHD to distinguish the underlying cognitive characteristics.

Those with ADHD have dysfunctions in the later stage of information processing. For example, individuals with ADHD show lower efficiency in selective attention in late information processing compared to the healthy controls and high perceptual load, which requires early selective attention to effectively eliminate the inefficiency of selective attention (Forster et al., 2014). Besides, deficient late selective attention suggests problems in executive functioning, especially response inhibition (Forster and Lavie, 2007, 2009).

While attentional problems have been repeatedly proven to exist in those with SCT (e.g., selective attention), mixed results were reported regarding the information processing that is affected by attention (Mueller et al., 2014; Barkley, 2018; Becker and Barkley, 2018; Kofler et al., 2019). For example, those with SCT showed impaired information processing including visual-perception, attention network, and processing speed (Camprodon-Rosanas et al., 2017; Wood et al., 2017; Jacobson et al., 2018; Tamm et al., 2018). However, several studies found no relation between SCT symptoms and processing speed, spatial memory, and response inhibition (Skirbekk et al., 2011; Bauermeister et al., 2012; Jarrett et al., 2017).

Despite there being no direct evidence, some research suggests the possibility of poor attention in early stages of information processing. First, dysfunction of early selective attention was found to be related to SCT symptoms such as slowness and confusion in thinking considering the impairment of visual-perceptual/spatial abilities: attention to detail (Huang-Pollock et al., 2005; Handy and Kam, 2015; Tamm et al., 2018). Second, a fMRI study found an association between increasing SCT symptoms and hypoactivity in the left superior parietal lobe, implying impaired function in receiving and encoding a great deal of visual input, seemingly related to impaired early information processing (Fassbender et al., 2015). Third, abnormal early selective attention was suggested in children with high SCT symptoms (Huang-Pollock et al., 2005). However, the study was conducted with groups of people with ADHD and controls (i.e., the result of SCT might be confounded by the presence of ADHD) and used only four items identifying SCT symptoms as a secondary analysis. Thus, it is essential to reconfirm the specific attentional problems of SCT in information processing and distinguish them from those of ADHD.

The present study applied the prominent theory called “load theory” to enhance the understanding of attention deficit in information processing in SCT. Load theory was originally proposed to solve the longstanding debate of early vs. late attentional selection in cognitive psychology (Maylor and Lavie, 1998; Lavie et al., 2004). Perceptual load, found to reduce distraction effectively in non-clinical population, has been used to investigate the deficiency of selective attention in each stage of information processing for those with ADHD compared to non-clinical population, suggesting effective interventions (Huang-Pollock et al., 2005; Remington et al., 2012; Forster et al., 2014). As it was helpful to investigate specific mechanisms of selective attention in individuals with ADHD, it would also be helpful to relate the symptoms of selective attention of SCT, apparently similar to that of ADHD, expressed as being “easily distracted” or “mentally confused,” to specific mechanisms in individuals with SCT (Murphy and Greene, 2017).

According to load theory, a key determinant of the ability to focus attention is whether the task being performed involves a high perceptual load sufficient to fill perceptual capacity. When tasks involve a low load (e.g., involving few items), it leaves the capacity that can spill over, resulting in involuntary processing of distractors. In this respect, low load tasks necessitate the ability of late selective attention to minimize interference, relying on executive mechanism and active inhibition, happening later than perceptual processes (Lavie, 1995). Conversely, when the task processing involves a high load (e.g., searching among many items), it uses up the available perceptual capacity, and therefore perception of distractors is reduced or even eliminated. Thus, higher levels of perceptual load engender more efficient early selective attention and make individuals stay focused on task-relevant stimuli. The deficit in early selective attention is related to the difficulty in distinction of the target-distractor, and low perceptual capacity (Swettenham et al., 2014).

In sum, although SCT symptoms result in lowering daily life functioning, the core problems of information processing of SCT have not been demonstrated yet. The results while studying cognitive symptoms have been confounded by various information processes including perceptual process, response selection, and partly due to the tasks used in each study focusing on different constructs and subject selection (VanRullen and Thorpe, 2001; Kofler et al., 2019). The present study attempted to focus on selective attention, which is one of the proven symptoms of SCT, and differentiate it from that in ADHD using load theory and a corresponding task (i.e., irrelevant distractor task). As the present study aimed to investigate the distinguished problem of SCT as an independent disorder, the study was conducted on individuals with SCT who does not show high level of ADHD symptoms, and on those with ADHD who does not show high level of SCT symptoms not to confound the results. In addition, there is no consensus on whether those with SCT and those with ADHD who do not show high levels of SCT symptoms have visuospatial working memory (VSWM) deficits, which impact their selective attention (Skirbekk et al., 2011; Bauermeister et al., 2012; Tamm et al., 2018). To deal with this, the present study investigated the difference of VSWM in individuals with SCT and ADHD from controls to explore the effect of VSWM.

Overall, the aim of the present study was to investigate the decreased efficiency of selective attention in early information processing in individuals with SCT and compare it to individuals with ADHD and controls. It is hypothesized that individuals with SCT will show a marked inefficiency in selective attention in early information processing compared to controls, while there should be no marked deficit in selective attention in late information processing, in accordance with the evidence of perceptual and attentional difficulties. However, individuals with ADHD will show a marked inefficiency of selective attention in late information processing compared to controls, while no deficit in selective attention in early information processing considering the distractibility and difficulty in response inhibition.

## Materials and Methods

### Participants and Screening

The sample size was calculated using the program G^*^Power 3.1 (Faul et al., 2007), that estimated a sample size of 66 participants as adequate for a design with repeated-measure analysis of variance (ANOVA), an alpha error probability of 0.05 (two-tailed), a power of 0.95, and a medium effect size ($\eta_{p}^{2}$ = 0.25).

Prior to the experiment, as an initial screening for SCT and ADHD, a total of 745 adults completed the Barkley Adult ADHD Rating Scale IV (BAARS-IV; Barkley, 2011). They were recruited through advertisements in online communities for individuals with attentional problems, and an internet bulletin board of several universities in Seoul, Korea. The participants consisted of females (68.7%) and males (29.5%), ranging from 18 to 55 years in age (*M* = 22.7, *SD* = 4.6).

Based on previous recommendations on the inclusion criteria (Barkley, 2012, 2013), a symptom threshold of 95th percentile or higher symptoms corresponding to five or more symptoms was used to identify SCT and four or more symptoms to identify ADHD. This threshold was coupled with self-reported impairment in one or more major life activities. In the experiment, all participants were interviewed with the structured clinical interview for DSM-5 (SCID-5; First et al., 2016) by a trained graduate student to determine eligibility. Those who had history of psychiatric disorders or related medical condition were excluded from the analysis of SCT group and control group. For the ADHD group, those who had history of psychiatric disorders other than ADHD were excluded from analysis. Control participants were randomly selected among those who did not show ADHD symptoms (lower level of ADHD compared to the mean value on the inattention and hyperactivity-impulsivity subscale of Barkley Adult ADHD Rating Scale) and SCT symptoms (lower level of SCT symptoms compared with the mean value on the SCT subscale of Barkley Adult ADHD Rating Scale and Adult Concentration Inventory). Since there is no clear inclusion criteria on SCT, we applied strict criteria: five or more symptoms of SCT, self-reported functional impairment due to attentional problems on BAARS-IV, and no diagnosis of psychiatric disorders. Participants who were color-blind were excluded because the color of elements in the attentional capture task played a role as a distractor.

Of the 87 participants, 14 participants were excluded; 8 participants were diagnosed with other disorders (e.g., depression, vasovagal syncope, and narcolepsy), 1 participant was on antidepressant medication, 3 participants did not follow instructions well, and 1 participant was excluded due to low accuracy (<60%) in the irrelevant distractor task (Forster and Lavie, 2016). Finally, a total of 73 individuals participated in the present study: SCT group (*n* = 24), ADHD group (*n* = 24), and control group (*n* = 25). The participants included males (38.36%) and females (61.64%) and ranged from 18 to 29 years in age (*M* = 22.04, *SD* = 2.59).

### Questionnaires

#### The Barkley Adult ADHD Rating Scale IV (BAARS-IV)

BAARS-IV was used to assess symptoms of SCT and ADHD and subsequently assign participants to groups. BAARS-IV is a validated tool to assess the levels of ADHD and SCT (Barkley, 2011). Since the Korean version of BAARS-IV has not been validated, BAARS-IV was translated into Korean by consulting a clinical psychologist with an expertise in attention problems; the scale was back translated into English with the aid of a bilingual interpreter. The appropriacy of the translation was evaluated by comparing the original and back translated versions; the content of several questions was revised accordingly. BAARS-IV contains 18 items that are consistent with DSM-5 criteria for ADHD and 9 items that target the symptoms of SCT (e.g., prone to daydreaming when I should be concentrating on something or working; easily confused; slow moving). Using a four-point Likert scale (1 = not at all; 2 = sometimes; 3 = often; 4 = very often), the participants responded to each item with reference to how often each statement best described their behavior in the past 6 months. The higher the BAARS-IV score of each subscale, the more attentional symptoms they experienced. In the present study, Cronbach's α values were 0.81, 0.88, and 0.84 for the subscales of ADHD inattention, ADHD hyperactive-impulse, and SCT, respectively.

#### The Adult Concentration Inventory (ACI)

ACI was used to confirm the differences in the level of SCT among groups. Originally developed as a new adult self-report measure of SCT (Becker et al., 2015), ACI was used in this study after the same translation procedure used for BAARS-IV. Among 16 items, 10 items of ACI were identified as optimal for the assessment of SCT symptoms in a validation study of 3,172 undergraduate students (Becker et al., 2018). Thus, the present study analyzed these 10 items. Items were rated on a four-point Likert scale (0 = not at all; 1 = sometimes; 2 = often; 3 = very often) with reference to the past 6 months. The higher the ACI score, the more SCT symptoms they experienced. Cronbach's α of the 10-item ACI scale was 0.89 in the validation study (Becker et al., 2018) and 0.86 in the present study.

#### The Beck Depression Inventory-Second Edition (BDI-II)

BDI-II was used to compare and control the level of depression among participants. It was developed to assess the levels of depression (Beck et al., 1996), and has been validated in Korean (Lim et al., 2014a). It includes 21 items associated with physical and cognitive symptoms of depression rated on a four-point Likert scale (0 = not at all; 1 = mildly; 2 = moderately; 3 = severely) with reference to the past 1 week. The higher the BDI-II score, the higher level of depression. Cronbach's α was 0.89 in the validation study and 0.91 in the present study.

#### The Beck Anxiety Inventory (BAI)

BAI was used to compare and control the level of anxiety among participants. It was developed to assess the levels of anxiety (Beck et al., 1988) and has been validated in Korean (Lim et al., 2014b). It includes 21 items related to physical and cognitive symptoms of anxiety rated on a four-point Likert scale (0 = not at all; 1 = mild; 2 = moderate; 3 = severe), with reference to the last week. The higher the BAI score, the higher the level of anxiety. Cronbach's α was 0.91 in the validation study and 0.91 in the present study.

### Behavioral Methods

#### The Irrelevant Distractor Task

The irrelevant distractor task was used to measure early vs. late selective attention. The task was developed based on load theory and optimized to represent the characteristics of visual salience and meaningfulness of distractor (Maylor and Lavie, 1998; Huang-Pollock et al., 2002; Forster and Lavie, 2008).

**Figure 1:**
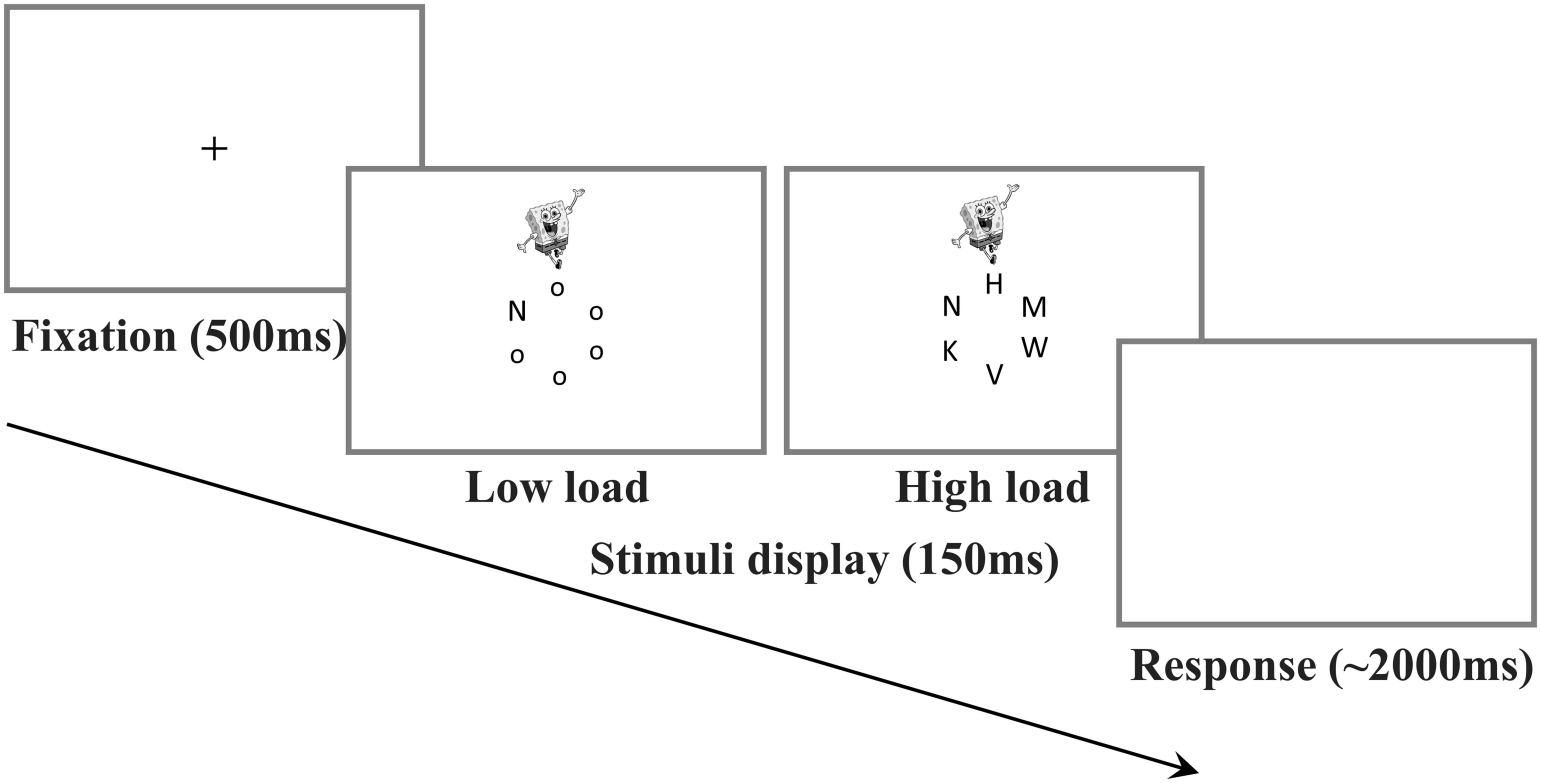
Trial example of the irrelevant distractor task. Note. The figures show the load condition (low vs. high) and the distractor condition (distractor vs. no-distractor) of the irrelevant distractor task. Both stimuli display of low and high load conditions include only distractor condition (Spongebob).

In each trial, participants were instructed to search the letter circle for a target letter (either X or N) and respond as fast as possible while still being accurate, ignoring any stimuli except for the letter search set. They were asked to respond using the numerical keypad by pressing the “0” key if the target was an X and the “2” key if the target was an N. Each trial began with a 500 ms presentation of a fixation cross, followed by a 150 ms presentation of six letters arranged to form a circle. Each trial ended either upon response, or after 2,000 ms if no response was made. A beep sounded for incorrect or missed responses (See Figure 1 for example trial display).

The load condition and distractor condition were investigated in the task. The load condition consisted of four levels, set size 1, 2, 4, and 6, which were for investigating the load effect. Set sizes refer to the number of letters shown in the display. Set size 4 and 6 had a relatively high perceptual load, called “high load,” and set size 1 and 2 had a relatively low perceptual load, thus called “low load.” Additionally, there were distractor conditions that were measured for selective attention. On 75% of trials (no-distractor baseline condition), no distractor was presented. On 25% of the trials (task-irrelevant distractor condition), a cartoon character was presented either above or below the letter circle.

The participants completed 3 slow example trials and 12 practice trials for each level of load prior to experimental trials. The example trials were made up of only no-distractor conditions, and the stimuli display remained until response. In the practice trials, no-distractor and distractor conditions were included, and the stimuli display disappeared after 150 ms, which was the same as in the experimental trials. If the participants achieved an accuracy of 65% in the practice trials, the participants then performed experimental trials, consisting of 8 blocks of 48 trials in the order of 1-2-4-6-4-2-6-1. An optional rest period was allowed between blocks. All combinations of load, target position, target identity, distractor position, and distractor identity were fully counterbalanced. The task lasted ~20 min.

The accuracy was calculated by the number of correct trials divided by the number of all trials × 100 (%). The mean reaction time (RT) to press keys for correct trials was calculated as a function of a group and experimental condition. The higher the RT, the higher the speed of information processing. Moreover, the inefficiency index of selective attention (distractor cost) in early and late information processing was calculated to investigate the performance level integrating the impact of accuracy and response time. It was calculated by subtracting the RT in distractor condition of the correct trials from the RT in no-distractor condition. Further details are described in the Results. The higher the inefficiency index, the lower the efficiency of selective attention.

#### The Corsi Block-Tapping Task

The Corsi block-tapping task was used to measure VSWM (Corsi, 1972; Kessels et al., 2000; the computerized version adapted by Mammarella et al., 2008). The Corsi block-tapping task was developed and widely used to assess short-term and working memory in the visuospatial domain. Several researchers have reported deficit in VSWM ability in individuals with SCT, and VSWM ability is one of the factors that influence the selective attention in information processing (Skirbekk et al., 2011; Bauermeister et al., 2012; Murphy et al., 2016). Thus, the Corsi block-tapping task was conducted to compare group differences in VSWM and to covariate the variable if there was a difference.

The task consisted of 16 trials, which gradually increased in length of 2 to 9 sequences. Participants were shown nine blue identical blocks randomly arranged on a display on a black background. In each trial, a sequence of blocks was highlighted by the color change of the block from blue to yellow for 1,000 ms. The participant was asked to click the same blocks in the same order as soon as the color change of the block ended. The length of the sequence increased gradually with one added block to the sequence after two trials. Self-corrections were permitted. The task terminated automatically if the participant failed to produce both sequences of equal length correctly. The task lasted about 3 min.

The digit span score and total score was acquired from the task. The digit span was measured as the length of the sequence in which at least one of the two trials was reproduced correctly. Total score was taken from the total number of correct block sequencing in all trials. The higher the digit span and total score, the higher the VSWM capacity.

#### Korean-Wechsler Adult Intelligence Scale-IV (K-WAIS-IV) Short Form

The Wechsler Adult Intelligence Scale-Fourth Edition (WAIS-IV) was developed as a measure of general intellectual functioning (Wechsler, 2008). In the present study, a brief version of K-WAIS-IV was used to see if there was a difference in intelligence among groups and control the difference. The Arithmetic (AR) and Information (IN) subtests of the K-WAIS-IV were used, as these subtests were reported to have the strongest correlation with the full scale intelligence quotient (IQ) in the K-WAIS-IV as a screening measure of intelligence (Hwang et al., 2012; Choe et al., 2014). The estimated full-scale IQ was calculated using suggested regression equations [54.762 + (2.330 × AR) + (2.151 × IN)] (Choe et al., 2014). The higher the estimated IQ, the higher their intelligence.

### Apparatus

For the behavioral task, participants were tested individually in a quiet room and they conducted the task at a viewing distance of approximately 60 cm from a 15-inch monitor with a resolution of 1,920 × 1,080 pixels. The stimuli in the irrelevant distractor task were presented electronically using the E-Prime 2.0 software (Psychology Software Tools Inc., 2017; Pittsburgh, PA, USA). All stimuli were presented on a black background, with all letter stimuli presented in light gray. In the irrelevant distractor task, the letter circle radius subtended 1.6° of visual angle, with target letters subtending 0.6° by 0.4°. In the set size 2, 4, and 6 conditions, non-target letters other than the target were randomly chosen from the set H, K, M, V, W, and Z. In the set size 1, 2, 4 conditions, the remaining non-target positions were occupied by small “o”s (0.15° by 0.12°). For example, the target letter (either X or N), three capital letters (e.g., H, K), and two small “o”s were displayed in the set size 4. The target letter (either X or N) and five capital letters without any “o” were displayed in the set size 6. On the distractor trials, a full-color cartoon image was presented 4.6° from fixation and subtended vertically 2.8°-4° and horizontally 2.8°-3.2° of visual angle. Each distractor image was drawn from the following set of cartoon characters: Superman, Spiderman, Spongebob Squarepants, Pikachu, Mickey Mouse, and Donald Duck (Forster and Lavie, 2008). The Corsi block-tapping task was programmed using Inquisit software 5.0 for windows (Millisecond Software, 2015, Seattle, WA, USA).

### Procedure

The participants were invited to the laboratory and given brief instructions on the procedure and their rights as research participants. They signed an informed consent form approved by the institutional review board of Chung-Ang University (No. 1041078-201910-HRSB-317-01). All participants were interviewed using the SCID-5 to determine eligibility for the experiment. Participants with no history of psychiatric disorders were interviewed with the brief version of WAIS-IV and completed the self-report questionnaires (BDI-II and BAI). Each participant was asked to sit and face the computer monitor from the distance of ~60 cm. Each participant performed irrelevant distractor task and the Corsi block-tapping task at a viewing distance of ~60 cm from a 15-inch monitor. The order of the tasks was counterbalanced. After all procedures were finished, the participants were debriefed on the study and each procedure and received 10,000 won (ca. 10 USD) as monetary reward. In debriefing, participants heard the aim of the study and each construct measured in the study. If participants wanted to get the results of the research, a brief report on individual results and general results of the experiment were provided. The entire experimental session took ~30 min.

### Data Analysis

For the group characteristics, one-way ANOVAs and chi-square tests were conducted to examine differences among groups in terms of sex, age, estimated IQ, VSWM span, and score, each subscale of BAARS-IV, ACI, BDI, and BAI. To investigate the accuracy and speed in early and late selective attention in information processing, a 3 (group: SCT, ADHD, control) × 2 (load: low, high) × 2 (distractor: distractor, no-distractor) repeated measures analysis was performed. A 3 (group: SCT, ADHD, control) × 2 (load: low, high) repeated measures analysis of ANOVA on the distractor cost was employed to compare group differences between the efficiency of selective attention in early and late information processing. When the three-way interaction was significant, analysis of group × load was conducted under each load to reveal the group differences with and without distractor under each load. Comparing means adjusted by Bonferroni *post-hoc* test and paired *t*-test were conducted for significant two-way interaction and main effect. All statistical data were analyzed using SPSS 25.0 for Windows.

## Results

### Group Characteristics

Table 1 shows group and clinical characteristics of participants. The results show significant differences among groups in the symptoms of ADHD and SCT. That is, there was a significant effect of groups for ADHD symptoms including inattention [*F*(2, 70) = 76.51, *p* < 0.01, $\eta_{p}^{2}$ = 0.69] and hyperactivity-impulsivity [*F*(2, 70) = 20.69, *p* < 0.01, $\eta_{p}^{2}$ = 0.37]. Specifically, the ADHD group had significantly higher ADHD symptoms compared to SCT and control groups. In addition, the ADHD group showed significantly higher SCT symptoms than the control group. There were significant effects of groups for SCT symptoms including SCT [*F*(2, 70) = 139.03, *p* < 0.01, $\eta_{p}^{2}$ = 0.80] in BAARS-IV, and ACI [*F*(2, 70) = 65.75, *p* < 0.01, $\eta_{p}^{2}$ = 0.65]. The SCT group had significantly higher SCT symptoms than ADHD and control groups. Additionally, it showed significantly higher ADHD symptoms than the control group. These results indicate that the groups were appropriately divided, although the SCT group and ADHD group reported higher attentional symptoms regardless of group division.

**Table 1:** Demographic and clinical characteristic for each group.

**Measure**	**SCT (1)** ** (*n* = 24)**	**ADHD (2)** ** (*n* = 24)**	**Control (3)** ** (*n* = 25)**	**Test** ** Statistics** ** (*F*/χ^2^)**	**Bonferroni**
Age (years)	21.08 (1.95)	23.17 (3.08)	21.92 (2.28)	4.23	
Sex (male/female)	9/14	6/18	13/13	3.31	
IQ	104.22 (7.34)	101.11 (15.53)	105.14 (6.35)	0.83	
BAARS-IV	18.13 (2.42)	21.21 (2.54)	13.08 (2.00)	76.51^*^	2 > 1 > 3
ADHD IN					
BAARS-IV	13.71 (2.85)	17.46 (6.07)	10.24 (1.36)	20.69^*^	2 > 1 > 3
ADHD H-I					
BAARS-IV	23.92 (1.69)	19.46 (3.19)	12.60 (2.06)	139.03^*^	1 > 2 > 3
SCT					
ACI	27.42 (3.31)	22.88 (4.21)	15.88 (3.06)	65.75^*^	1 > 2 > 3
BDI-II	16.54 (8.05)	12.13 (9.01)	6.16 (5.15)	11.68^*^	2, 1 > 3
BAI	10.79 (7.99)	9.63 (9.65)	3.64 (3.86)	6.40^*^	2, 1 > 3
VSWM span	6.75 (0.99)	6.71 (1.16)	7.04 (1.02)	0.72	
VSWM score	69.38 (21.59)	70.04 (24.34)	75.52 (19.30)	0.59	

Note. Mean (standard deviation);

* *p < 0.01; 1: control, 2: SCT, 3: ADHD; Age: years, SCT, sluggish cognitive tempo; ADHD, attention-deficit/hyperactivity disorder; BAARS-IV, Barkley Adult Attention-Deficit/Hyperactivity Disorder Rating Scale IV; IN, Inattentive; H-I, hyperactive and impulsive; ACI: Adult Concentration Inventory; BDI-II, Beck Depression Inventory-II; BAI, Beck Anxiety Inventor; IQ, Intelligence Quotient estimated by brief version of Wechsler Adult Intelligence Scale- IV; VSWM, Visuospatial Working Memory measured in Corsi block-tapping task; Test Statistics (F), results of the omnibus F-test; Test Statistics (χ^2^), results of the chi-square test, When the F test was significant, the results of pairwise group comparison using Bonferroni are shown as well*.

Regarding other clinical symptoms, there were significant effects of groups for BDI-II [*F*(2, 70) = 11.68, *p* < 0.01, $\eta_{p}^{2}$ = 0.25] and BAI [*F*(2, 70) = 6.40, *p* < 0.01, $\eta_{p}^{2}$ = 0.16]. The SCT and ADHD groups showed higher levels of anxiety and depressive symptoms than the control group. However, there were no significant differences in VSWM span [*F*(2, 70) = 0.72, *n.s*.] and total score [*F*(2, 70) = 0.59, *n.s*.] in Corsi block-tapping task and the estimated IQ [*F*(2, 70) = 0.83., *n.s*.] among the groups. These results indicate that the groups did not differ in age, sex, intelligence, and VSWM.

When it comes to demographic characteristics, there were no significant differences in the mean age [*F*(2, 70) =.4.23, *n.s*.] and the proportion of sex [χ^2^ (2) = 3.31, *n.s*.] among the groups.

### Accuracy

The accuracy was compared to consider the relationship between accuracy and response time before analyzing the efficiency of selective attention among groups. To do this, a 3 (groups: SCT, ADHD, Control) × 2 (distractor: distractor, no distractor) × 2 (load: low, high) mixed-model ANOVA was performed on accuracy (see Table 2). There was a significant two-way interaction between group × load [*F*(2, 70) = 3.84, *p* < 0.05, $\eta_{p}^{2}$ = 0.10]. However, when *post-hoc* tests using Bonferroni correction were performed to explore the significant interaction, there was no significant group difference in each load nor load differences in each group [*F*(2, 70) <2.27, all *n.s*.]. In addition, there was a significant main effect of distractor [*F*(2, 70) = 7.34, *p* < 0.001, $\eta_{p}^{2}$ = 0.105] and load [*F*(2, 70) = 76.057, *p* < 0.001, $\eta_{p}^{2}$ = 0.52], indicating that manipulation of load and distractor was effective. There was no significant three-way interaction within group × distractor × load, nor two-way interactions between effects of distractor × load, and load × *group*, or main effect of group [*F*(2, 70) <2.22, all *n.s*.].

**Table 2:** Mean (SD) of Accuracy and response time under each load and distractor condition for groups in irrelevant distractor task.

**Load condition**	**Distractor condition**	**SCT** ** (*n* = 24)**	**ADHD** ** (*n* = 24)**	**Control** ** (*n* = 25)**
**Accuracy**				
High	No-distractor	96.279 (3.078)	94.877 (5.094)	94.951 (5.623)
	Distractor	89.583 (4.556)	86.111 (10.846)	91.548 (7.610)
Low	No-distractor	96.275 (2.077)	96.546 (2.723)	95.622 (3.888)
	Distractor	90.514 (6.185)	89.410 (7.180)	92.135 (5.024)
**Response time**				
High	No-distractor	477.408 (60.174)	491.543 (66.060)	432.837 (61.329)
	Distractor	494.724 (74.403)	482.199 (63.076)	441.262 (60.180)
Low	No-distractor	327.944 (32.058)	335.848 (30.536)	313.007 (30.783)
	Distractor	346.442 (40.234)	352.721 (38.920)	324.617 (28.310)

Note. Mean (standard deviation); *p < 0.05; SCT group, Sluggish cognitive tempo group; ADHD group, Attention-deficit/hyperactivity disorder group; High load condition is hypothesized to measure early selective attention. Low load is hypothesized to measure late selective attention.

These results indicate that the accuracy decreased when the load increased and the distractor was presented in the task, whereas the accuracy was not influenced by group differences. Thus, there was no need to combine the level of accuracy to the calculation of the efficiency index and the effect of accuracy could be controlled simply by including only correct responses.

### Response Time

To examine the differences of the speed of selective attention in each stage of information processing among groups, a 3 (group: SCT, ADHD, Controls) × 2 (distractor: distractor, no distractor) × 2 (load: low, high) mixed-model ANOVA was performed on RT (see Table 2). There was a significant three-way interaction of group × distractor × load [*F*(2, 70) = 4.20, *p* < 0.05, $\eta_{p}^{2}$ = 0.15]. In addition, there were significant two-way interactions between group × distractor [*F*(2, 70) = 3.58, *p* < 0.05, $\eta_{p}^{2}$ = 0.09] and load × distraction [*F*(2, 70) = 6.61, *p* < 0.05, $\eta_{p}^{2}$ = 0.086]. To explore the significant three-way interaction to investigate our hypothesis, two-way interactions between group × distractor in each load were analyzed and described.

**Figure 2:**
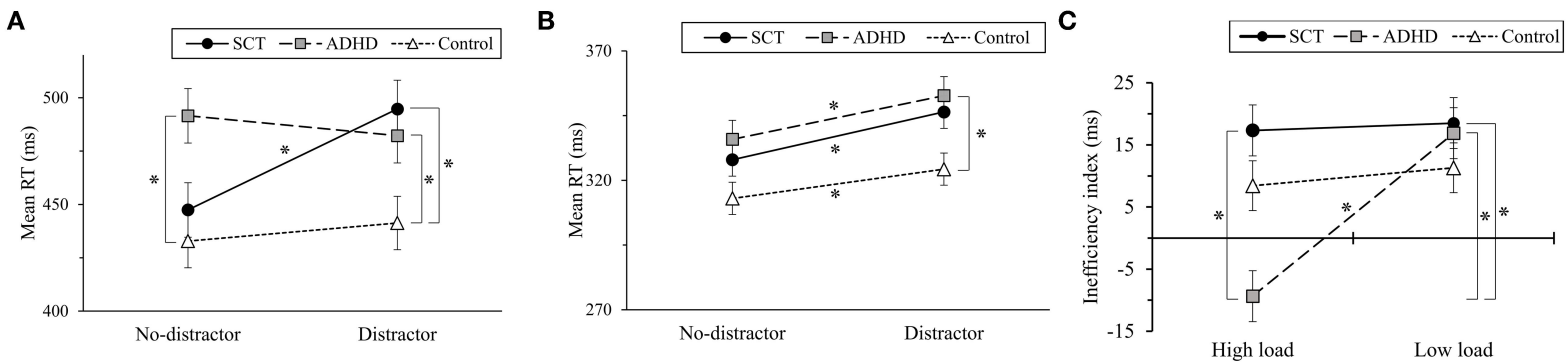
**(A)** Comparison of mean RT for groups in high load. **(B)** Comparison of mean RT for groups in low load. **(C)** Comparison of inefficiency index (distractor cost) for groups in low and high load. Note. High load condition is hypothesized to measure early selective attention. Low load condition is hypothesized to measure late selective attention. Error bars represent standard error of the mean. SCT, sluggish cognitive tempo group; ADHD, attention-deficit/hyperactivity disorder group. No-distractor, no-distractor condition; Distractor, distractor condition; Inefficiency index (distractor cost), RT in distractor condition—RT in no-distractor condition (ms). *p < 0.05.

Under high load, there was a significant interaction of group × distractor [*F*(2, 70) = 5.31, *p* < 0.01, $\eta_{p}^{2}$ = 0.132]. To examine the group × distractor interaction under high load, the differences of RT for the groups in no-distractor condition and distractor condition were analyzed using Bonferroni correction. In no-distractor condition, there was a significant difference in RT among groups [*F*(2, 70) = 4.41, *p* < 0.05, η^2^ = 0.11]. The ADHD group (*M* = 491.54, *SD* = 66.06) showed significantly high RT compared to that of the control group (*M* = 432.84, *SD* = 61.33) (*p* < 0.05). In distractor condition, there was also a significant difference of RT among groups [*F*(2, 70) = 4.41, *p* < 0.05, $\eta_{p}^{2}$ = 0.11]. The SCT group (*M* = 494.73, *SD* = 74.40) and the ADHD group (*M* = 482.20, *SD* = 63.08) showed significantly high RT compared to the control group (*M* = 441.26, *SD* = 60.18) [*p* < 0.05]. When split into groups and analyzed using a paired samples *t*-test to examine the occurrence of early selective attention in each group under high load, there was no significant effect of distractor in the ADHD group [*t*(23) = −1.48, *n.s*.] and control group [*t*(24) = 1.74, *n.s*.]. However, there was a significant effect of distractor in SCT group [*t*(23) = −2.74, *p* < 0.05]. The SCT group showed higher RT in distractor condition (*M* = 494.72, *SD* = 74.40) compared to no-distractor condition (*M* = 477.41, *SD* = 60.17) (Figure 2A).

Under low load, there were significant main effects of group [*F*(2, 70) = 4.16, *p* < 0.05, $\eta_{p}^{2}$ = 0.11] and distractor [*F*(2, 70) = 43.72, *p* < 0.01, $\eta_{p}^{2}$ = 0.38]. However, there was no significant interaction between group × distractor [*F* (2, 70) = 0.87, *n.s*]. All participants showed increased RT under the distractor condition compared to no-distractor condition regardless of their group. Moreover, *post-hoc* using Bonferroni revealed that the ADHD group (*M* = 344.28, *SD* = 32.73) showed higher RT than the control group (*M* = 318.28, *SD* = 28.28)(*p* < 0.05). These findings show that all participants across groups showed a slowed speed in the late selective attention, and individuals with ADHD took a longer time to focus attention than controls in late information processing (Figure 2B).

These findings show that there was no significant difference between the SCT group and the controls, and only ADHD showed slower RT than the controls when with distractor in low load condition. Conversely, in high load, the SCT group needed more time to select where to focus on when with distractor compared to the controls. Moreover, the difference of early selective attention occurred in those with SCT, but not in those with ADHD and the controls. It means that high perceptual load could not eliminate distractibility in those with SCT, while others succeed to discriminate task-related stimuli among distractors under high load.

### Inefficiency Index of Selective Attention (Distractor Cost)

The inefficiency index (distractor cost) was calculated to investigate the level of inefficiency of selective attention in early and late information processing. Since there was no significant difference of accuracy among groups, the inefficiency index was calculated by subtracting RT on the no-distractor condition of correct trials from RT on the distractor condition. To investigate the inefficiency of early and late selective attention, a 3 (group: SCT, ADHD, control) × 2 (load: low, high) repeated measures ANOVA on the inefficiency index was performed. There was a significant interaction between group × load [*F*(2, 70) = 4.20, *p* < 0.05, $\eta_{p}^{2}$ = 0.11].

Regarding the differences of the group in high and low load, *post-hoc* analysis using Bonferroni correction was conducted. While there was no main effect of group under low load [*F*(2, 70) = 0.87, *n.s*.], there was a significant group difference under high load [*F*(2, 70) = 5.31, *p* < 0.01, η^2^ = 0.13]. The SCT group (*M* = 17.32, *SD* = 31.01) showed a significantly lower efficiency compared to the ADHD group (*M* = −9.34, *SD* = 31.02) [*p* < 0.05]. This result indicates that individuals with SCT showed lower efficiency of early selective attention compared to those with ADHD. In addition, the contrast test comparing all conditions using Bonferroni correction revealed that the SCT group under low load condition (*M* =18.50, *SD* = 17.57) also showed lower efficiency compared to the ADHD group under high load condition (*p* < 0.01).

The *post-hoc* test using split by group showed that there was no significant effect of load in the SCT group [*F*(2, 70) = 0.26, *n.s*] and the controls [*F*(2, 70) = 0.03, *n.s*], while there was a significant effect of load in the ADHD group [*F*(2, 70) = 11.49, *p* < 0.01, $\eta_{p}^{2}$ = 0.33]. Specifically, the ADHD group showed lower efficiency in low load (*M* = 16.87, *SD* = 24.67) than in high load (*M* = −9.34, *SD* = 31.02). It indicates that those with SCT and controls showed similar levels of inefficiency in early and late information processing, while those with ADHD had a lower efficiency in late information processing which improved in early information processing (Figure 2C).

There were also significant effects of group [*F*(2, 70) = 3.57, *p* < 0.05, $\eta_{p}^{2}$ = 0.093] and load [*F*(2, 70) = 6.613, *p* < 0.05, $\eta_{p}^{2}$ = 0.086]. The SCT group (*M* = 17.91, *SD* = 18.72) showed lower efficiency compared to the ADHD group (*M* = 3.77, *SD* = 20.66). Overall, participants showed lower efficiency in low load (*M* = 15.50, *SD* = 20.06) compared to high load conditions (*M* = 5.51, *SD* = 30.55).

In conclusion, the attentional characteristics of SCT could be distinguished in the efficiency and speed of early selective attention. Individuals with SCT showed low efficiency and slow speed in early selective attention (i.e., under high load). By contrast, the ADHD group showed lower efficiency of late selective attention compared to early selective attention (i.e., under low load than high load) and slow speed in late information processing.

## Discussion

This study examined the speed and efficiency of selective attention in early and late information processing among adults with SCT and ADHD. The present study demonstrated two key findings. First, the attentional problem of SCT was different from that of ADHD since individuals with SCT showed lower efficiency in early selective attention compared to those with ADHD. Second, individuals with SCT showed low efficiency and slow speed of selective attention in early information processing and did not show a significant attentional problem in late information processing.

The major finding is that individuals with SCT had a different mechanism of selective attention from those with ADHD. In addition, individuals with ADHD showed a slower speed and lower efficiency in late selective attention compared to controls, and the high perceptual load effectively eliminated the inefficiency of selective attention. However, individuals with SCT showed low efficiency in early selective attention, which means that the high perceptual load could not alleviate the inefficiency of selective attention. This implies that individuals with distractibility could be distinguished as SCT or ADHD depending on the presence of the problem of early selective attention.

Another major finding is that those with SCT showed low efficiency and slow speed in early selective attention in the present study. Under high load, the SCT group responded much slower to the target compared to the controls in distractor condition. Moreover, the SCT group spent more time neglecting distractors compared to the ADHD group under high load, evidencing problems in early selective attention. This result was consistent with the hypothesis of the present study and previous research (Huang-Pollock et al., 2005; Mueller et al., 2014). Results indicate that individuals with SCT might not show difficulty in normal situations, but they show apparent difficulty especially in a situation requiring the ability of early selective attention where it is complex or gives a lot of stimulation.

As early selective attention is related to the process of automatic monitoring and evaluation of environment, it may take a long time for those with SCT to perceive and focus on academic (e.g., mathematical formula) and social stimuli which is normally perceived quite fast and easily by others (Bauermeister et al., 2012). This problem could be represented as the symptom of mind-wandering, internal distraction, or mental confusion (Mikami et al., 2007; Carretié, 2014). The symptoms might make them feel left behind in academic or social situations and eventually could lead them to withdraw socially and not actively participate in goal-oriented activities (Becker and Barkley, 2018). In addition, the deficiency in selective attention might at least partially contribute to the depression or anxiety symptom they experience, as poor attentional control is suggested to increase vulnerability of emotional disorders (Quigley et al., 2017; Becker et al., 2019).

The results also support our hypothesis and reconfirm that those with ADHD have a disruption of later attention mechanisms, including the efficiency of executive cortical control (Huang-Pollock et al., 2005; Sonuga-Barke, 2005; Forster et al., 2014). Consistent with previous studies, the attentional problems of ADHD result from difficulties of response inhibition and vulnerability to external stimuli in boring situations (Sonuga-Barke, 2005). Therefore, facilitating early selection with high perceptual load can compensate for executive control deficits that otherwise lead to increased distraction in ADHD.

There are several things to note in the interpretation of results of the present study. First, the results do not exclude other problems of attention of ADHD. Participants with ADHD showed higher inefficiency under low load than high load. Therefore, it can be inferred that those with ADHD have difficulty with late selective attention. However, the results also showed that those with ADHD took more time compared to the controls regardless the presence of a distractor except in the no-distractor condition of low load. It may imply the problem of general slow processing speed, not restricted to late selective attention.

Second, it is worth integrating the present results with various models. The present study was conducted based on the load theory to demonstrate the different aspect of attentional problem, specifically “distractibility,” shown in those with SCT and ADHD (Mogg et al., 2004; Forster and Lavie, 2008, 2016). The prominent model of ADHD pathology is characterized by underlying problem of “executive function,” especially “response inhibition” and the results of the study support the model (Sonuga-Barke, 2005). In addition, there are other findings of problems of ADHD, such as processing speed and automatic attention (Caprì et al., 2019). For example, a recent theory called refined theory of automaticity suggested that those with ADHD show deficit of automatic attention as well as deficit of controlled attention, which is analogous to early selective attention and late selective attention in the present study (Capri et al., 2020). As mentioned above, those with ADHD may present deficit of overall attention or processing speed, not confined to a problem of late selective attention. However, most of the prior studies did not distinguish individuals with ADHD from those with SCT and did not control the effect of SCT, so the results might be mixed due to symptoms of SCT within the ADHD group. Thus, it is recommended for future studies to compare the selective attention of SCT and ADHD in the framework of various theories and to allow us to distinguish their attention problems more clearly.

The present study used the inefficiency index (distractor cost) to integrate the accuracy and RT of performance results. Since there was no significant difference between groups of accuracy in the present study, only correct trials were included in subsequent analysis of RT and inefficiency index to control the effect of accuracy. The inefficiency index of correct trials would be appropriate as this study aimed to identify the characteristics of SCT including mental confusion and sluggishness while responding to targeted activities in daily life.

Interestingly, the SCT group and the ADHD groups did not show any difference in performing VSWM task with the control group. Previous studies have shown mixed results on the relevance of SCT or ADHD to working memory (Skirbekk et al., 2011; Bauermeister et al., 2012; Tamm et al., 2018). However, it is found that working memory tends to correlate with the accuracy of attentional control, not the inefficiency of attentional control (Draheim et al., 2019). It is evidenced by several researches that SCT and ADHD are more related to variability than to the performance score of working memory (Skirbekk et al., 2011; Willcutt et al., 2014). Therefore, those with SCT and ADHD seem to be related to the problem of low efficiency or slow speed of selective attention rather than accuracy of it. It raises one possibility of therapeutic perspective that those with SCT and those with ADHD can make the most of their potential abilities if provided sufficient time to do so.

SCT can also be explored as a transdiagnostic or dimensional concept as well as a categorical disorder. In the present study, the SCT group reporting low symptoms of ADHD showed a low efficiency in early selective attention. In a previous study, high SCT group showed abnormality in early information processing, when not controlling the presence of ADHD symptoms (Huang-Pollock et al., 2005). Taken together, it turns out that SCT is related to deficit in early selective attention regardless of the levels of ADHD symptoms (Lovett et al., 2020; Wood et al., 2020). Thus, SCT would be understood as a slow cognitive process, especially early selective attention, which develops through independent or interactive causes and developmental pathways, for example, other cognitive systems, the negative balance system, and arousal/regulatory systems in a Research Domain Criteria (RDoC) framework (Becker and Willcutt, 2019). Further research would boost the understanding of the nature of SCT that views it from various perspectives and links it to other related factors.

Considering the results, different interventions could be suggested for those with SCT and ADHD (Forster et al., 2014). In an academic setting, SCT can be helped by decreasing perceptual load, so that they are not too overwhelmed, making it easier to select what to focus on (e.g., educational materials with simple font and suggestion of brief summaries). In contrast, those with ADHD can be helped by increasing perceptual load, so that they do not use their remaining resource on other external or internal stimuli (e.g., educational materials covered with several colors) (Forster et al., 2014). Regarding psychosocial functions, it can be suggested that those with SCT might feel more manageable in small groups, and it might be helpful to provide individual or small-group psychotherapy. In addition, they could benefit from social skills training, which presents a few social cues at first and gradually increases the number of social cues being processed. On the other hand, group therapy including more individuals (e.g., dialectical behavioral therapy based on group program) could help those with ADHD focusing on the therapy and alleviate their symptoms (Philipsen et al., 2010).

Some limitations need to be considered when interpreting the results of this study. First, the SCT and ADHD groups showed more attentional symptoms than the controls. It is not surprising that the SCT group showed higher ADHD symptoms and the ADHD group showed higher SCT symptoms compared to controls since both report attentional problems. The effect of SCT and ADHD on selective attention could be investigated more explicitly if a future study matches the levels of other attentional symptoms to the controls when forming each group. Second, the aim of the study was to investigate the core problems of SCT, cognitive symptoms, and distinguish these from those of ADHD. Even so, individuals with SCT report not only cognitive symptoms, but also clinically significant distress in academic and socioemotional function, which seems to be related to be perceptual/cognitive problems. Future research is needed to explore the association of early selective attention and factors interfering well-being, and their relative contributions in individuals with SCT. Third, it would be helpful to investigate the specific attentional process in those with SCT, for example, overt attention as well as covert attention. Although the present study attempted to distinguish the unique attentional problem that people with SCT have in information processing, it might have more power when demonstrated with direct measurement such as eye-movement. Fourth, even though the sample size of the present study was enough, which means more than 66 participants calculated by G^*^power, further study replicating the experiment is needed to generalize the results of the present study.

In summary, the present study provided empirical evidence for the deficiency of early selective attention in individuals with SCT, elaborating on the problems in information processing. Individuals with SCT show slower speed in selecting what to focus on in early information processing. Moreover, this study distinguished different problems of those with SCT from those with ADHD and how the difference manifests in early information processing. Those with SCT suffered from attention selection in the early stage, while those with ADHD suffered from attention selection in the late stage of information processing. The present study can suggest a possible method for differentiating the information processing of those with SCT and ADHD, and possible interventions that would be appropriate for each symptom.

## Data Availability Statement

The raw data supporting the conclusions of this article will be made available by the authors, without undue reservation.

## Ethics Statement

The studies involving human participants were reviewed and approved by Chung-Ang University. The patients/participants provided their written informed consent to participate in this study.

## Author Contributions

YP and J-HL: conceived the experiment. YP: designed the experimental task, participants' data acquisition, and data analysis. YP and J-HL: data interpretation, drafting of the manuscript. All the authors revised the manuscript critically and gave the final approval of the version to be published.

## Conflict of Interest

The authors declare that the research was conducted in the absence of any commercial or financial relationships that could be construed as a potential conflict of interest.
